# First report of a successful surgical management of left atrial myxoma coexisting with pulmonary squamous cell carcinoma and thymic cyst

**DOI:** 10.1186/s40001-024-01974-6

**Published:** 2024-07-18

**Authors:** Yichen Li, Mi Tang, Qin Wu, Jinfu Yang, Wangping Chen

**Affiliations:** Department of Cardiovascular Surgery, The Second Xiangya Hospital, Central South University, Middle Renmin Road 139, Changsha, 410011 China

**Keywords:** Cardiac tumor, Lung cancer, Myxoma, Thymic cyst, Surgical management

## Abstract

**Background:**

Primary cardiac tumors, while rare, present significant clinical challenges due to their diverse pathology and presentation. Lung cancer frequently metastasizes to the heart; however, cases involving primary cardiac tumors of different origins alongside primary lung cancer are exceedingly rare in the literature.

**Case presentation:**

We report the case of a 53-year-old female who presented with hemoptysis and was subsequently diagnosed with a left atrial myxoma, pulmonary squamous cell carcinoma, and a thymic cyst. This coexistence of multiple non-homologous tumors in a single patient is exceedingly rare.

**Conclusion:**

This case underscores the complexity of diagnosing and managing patients with multiple distinct tumors. The simultaneous occurrence of a primary cardiac myxoma, pulmonary squamous cell carcinoma, and thymic cyst is unprecedented, providing valuable insights for future clinical practice.

## Introduction

Cardiac tumors, though uncommon, present significant clinical challenges due to their diverse pathology and clinical presentation [1]. These tumors are classified into primary cardiac tumors, which originate within the heart, and secondary cardiac tumors, which metastasize from other primary sites. Primary cardiac tumors are notably infrequent, with an incidence much lower than secondary tumors, which are a hundred times more common [2, 3]. Among primary cardiac tumors, benign myxomas are the most prevalent, especially in the left atrium, comprising roughly 75% of cases. Despite their benign nature, myxomas can cause serious clinical problems, including intra-cavitary obstruction, embolic events, and systemic symptoms [4–7]. In contrast, secondary cardiac tumors often result from metastases, with lung cancer being a significant source. The incidence of cardiac metastasis in lung cancer patients ranges from 15 to 35% [6, 8], with pathways including direct invasion, lymphatic spread, and hematogenous dissemination. These metastases can lead to substantial morbidity and mortality, primarily due to complications like atrial fibrillation [9] and sustained ventricular tachycardia [10].

The coexistence of multiple non-homologous tumors in a single patient is exceedingly rare. This case report details the unique presentation and surgical management of a 53-year-old female diagnosed with a left atrial myxoma, pulmonary squamous cell carcinoma, and a thymic cyst. The simultaneous occurrence of these distinct pathological entities has not been previously documented, underscoring the complexity and clinical significance of this case.

## Case report

**Fig. 1:**
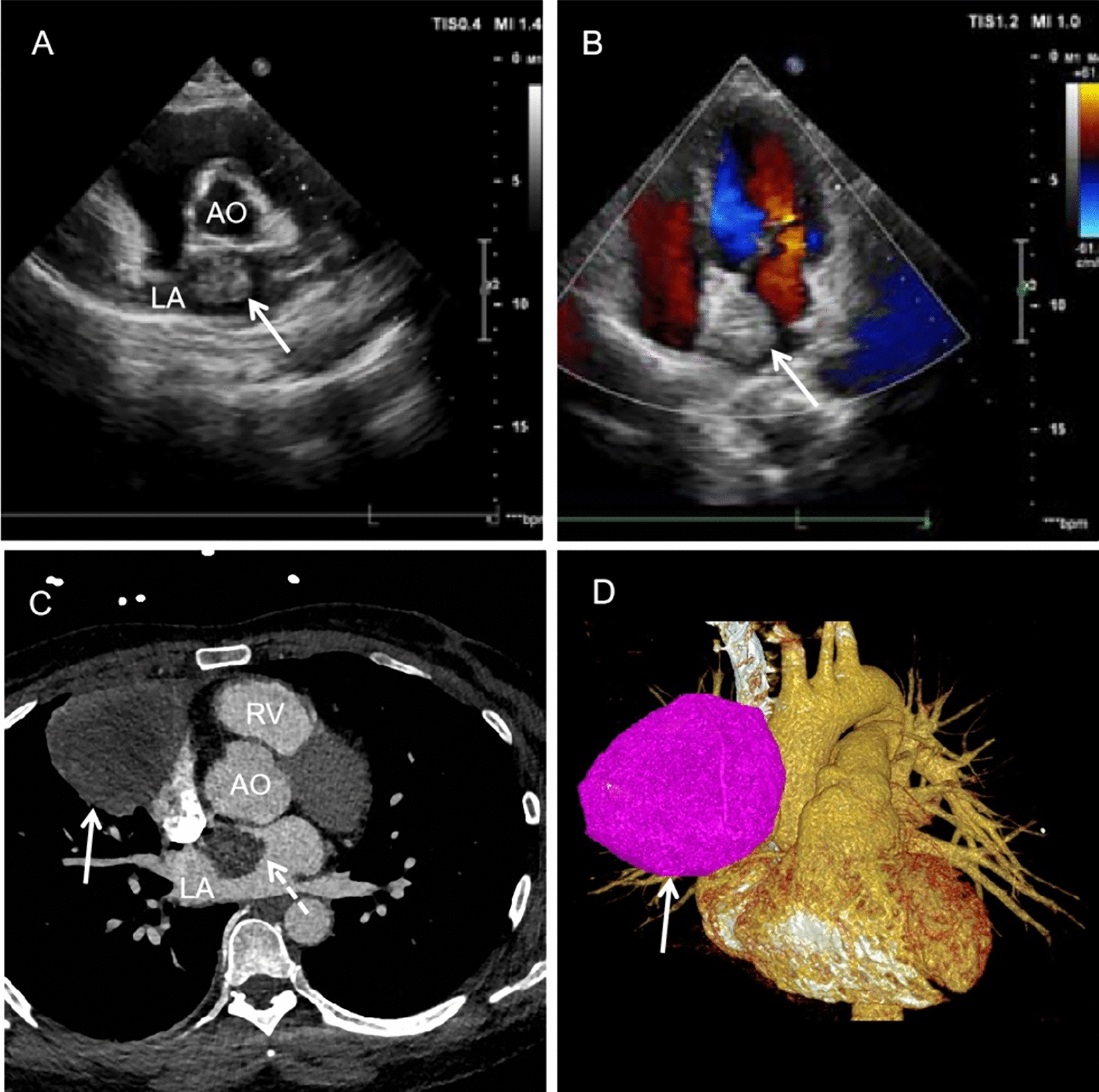
**A** Cardiac echo and color Doppler blood flow image (**B**) from left atrium angle, white arrow indicates the tumor. **C** Contrast-enhanced CT angiography image from transversal axis, white arrow indicates the right anterior mediastinum mass, white dotted arrow indicates the left atrium tumor. **D** 3D reconstruction image, white arrow indicates the right anterior mediastinum mass. *LA* left atrium, *AO* aortic, *RV* right ventricle

A 53-year-old female was referred to our hospital with exertional chest tightness for 1 month and hemoptysis for 1 week. The patient had no family history of hypertension, no history of chest trauma, or other cardiac health comorbidities. Physical examination revealed no significant clinical findings except for a slightly accentuated P2. Transthoracic echocardiography revealed a significant mass in the left atrium with the basilar part attached to the atrial septum (Fig. 1A and B). Contrast-enhanced CT angiography showed a well-defined mass with soft tissue density within the left atrium and a mass in the right anterior superior mediastinum, which was blood-supplied by the right superior pulmonary artery branch (Fig. 1C and D). A whole-body PET-CT scan showed no space-occupying lesions in other organs.

**Fig. 2:**
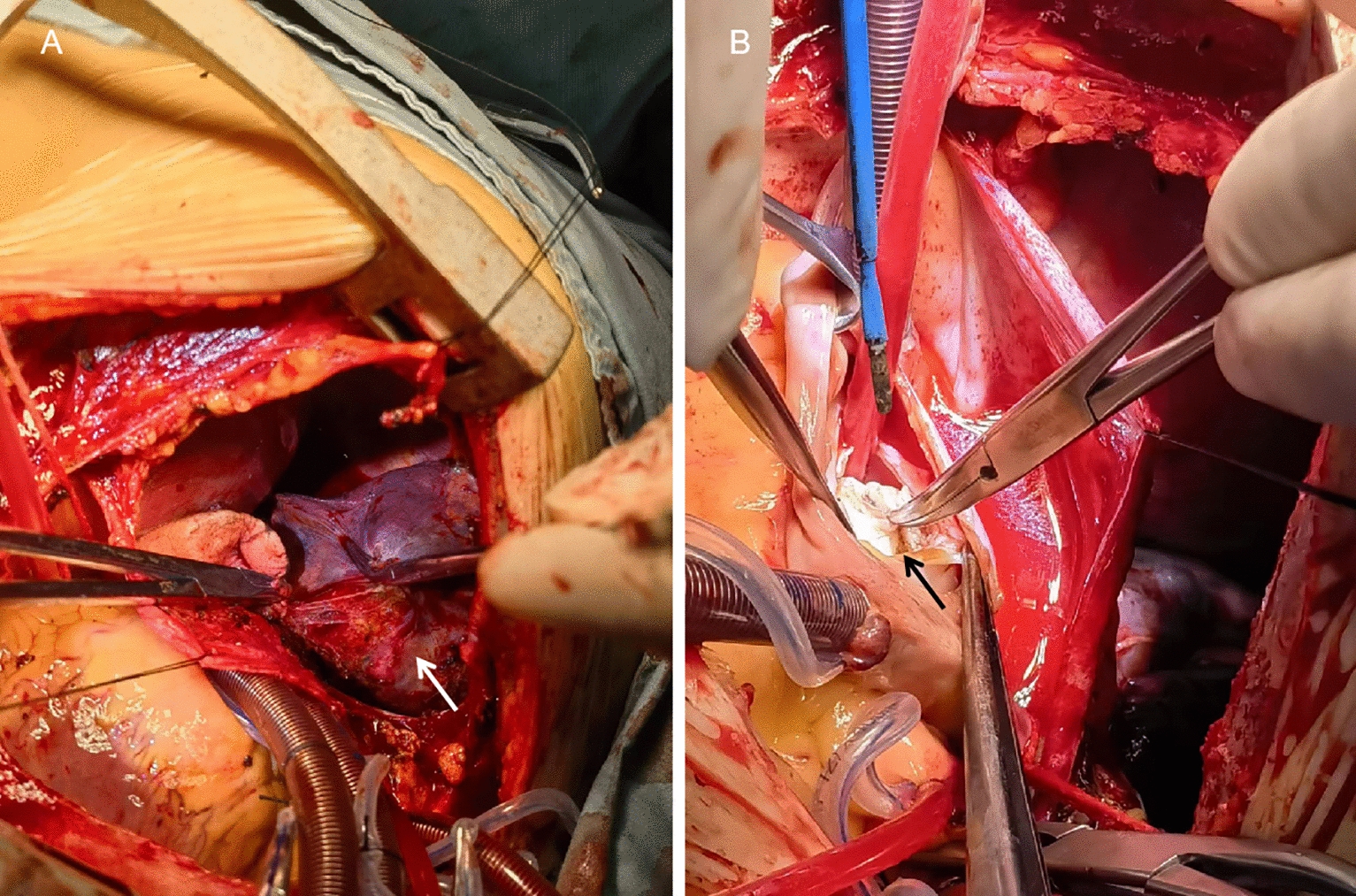
Surgical field of the open-chest operation. A White arrow indicates the right lung mass B Black arrow indicates the left atrium tumor

Upon admission and after thorough discussion among the medical team, the patient underwent an open thoracotomy. A standard median sternotomy incision was made to access the surgical area. In the anterior mediastinum, a thymic mass measuring 30 × 40 mm was observed. It was characterized by its soft, cystic nature and contained milky white fluid. Additionally, a mass measuring 100 × 80 mm was found in the right middle lung lobe. It had a well-defined capsule and was tightly adherent to the right chest wall, but not to the pericardium (Fig. 2A). Cardiopulmonary bypass was established by cannulating the aorta, superior vena cava, and inferior vena cava. After opening the right atrium and septum, a mass was discovered in the left atrium. It had a broad base and was attached to the mid-to-lower part of the atrial septum (Fig. 2B).

**Fig. 3:**
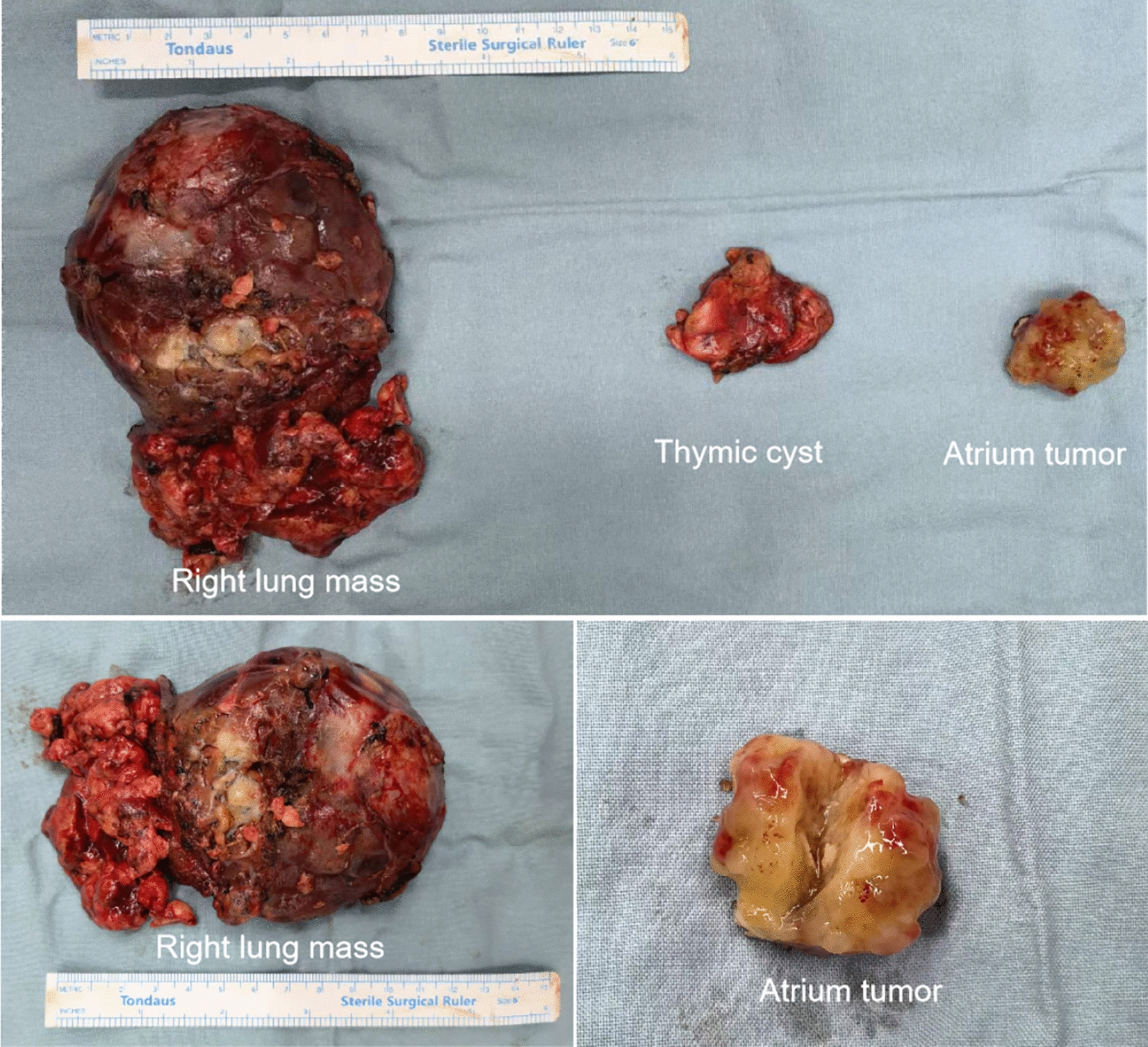
Removed right lung mass, thymic cyst and left atrium tumor

This mass measured approximately 30 × 35 mm and appeared brown and translucent with a firm texture. It was partially encapsulated. All three masses, the thymic cyst, right middle lung mass, and left atrium tumor, were completely removed (Fig. 3). And no tumors were found in any other cardiac cavities.

**Fig. 4:**
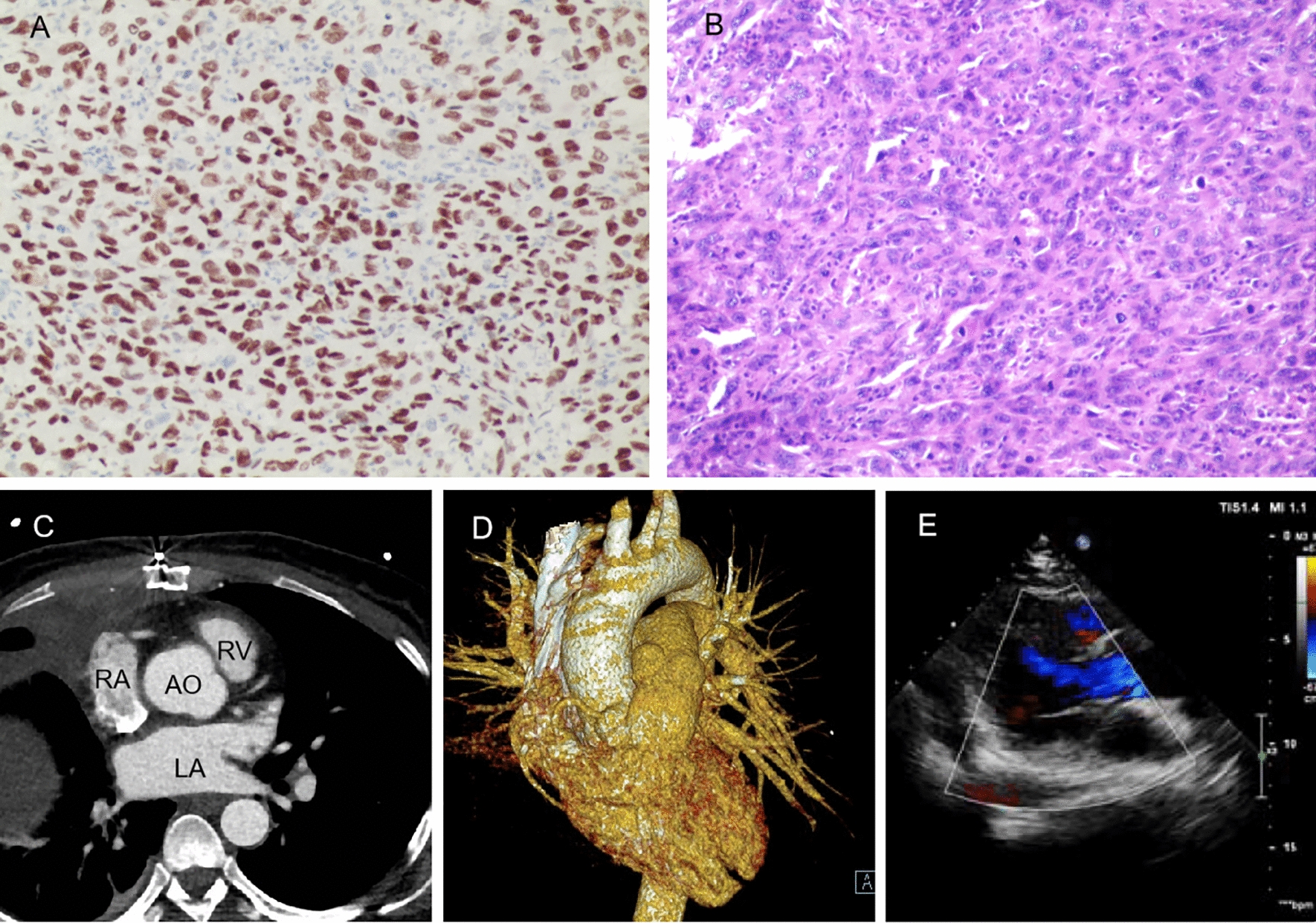
P40 staining (**A**) and HE staining (**B**) of the right lung mass, indicating poorly differentiated squamous cell carcinoma, 100× for **A** and **B**. Contrast-enhanced CT angiography (**C**) and 3D reconstruction (**D**) images post-operative show complete resection of the masses and no recurrence. **E** Cardiac echo post-surgery shows total remove of the left atrium tumor and no hemodynamic abnormality. *LA* left atrium, *RA* right atrium, *AO* aortic, *RV* right ventricle

Pathological examination and immunohistochemical analysis were conducted on the resected masses to determine their nature and origin. The mediastinal thymic mass had a grayish-yellow cut surface. Microscopic examination revealed fibrous cyst wall-like tissue lined by ciliated columnar epithelium, with thymic tissue observed around the cyst wall, suggesting a thymic cyst. The right middle lung mass was partially encapsulated, and upon sectioning, necrosis was observed. Histopathological analysis indicated a malignant tumor consistent with poorly differentiated squamous cell carcinoma, with focal areas exhibiting sarcomatous carcinoma characteristics. Under the microscope, extensive necrosis and significant cellular atypia were observed, with frequent mitotic figures. Immunohistochemical staining (Fig. 4A and B) supported the diagnosis of poorly differentiated squamous cell carcinoma with the following results: CK (+), TTF-1 (−), Napsin A (−), CD56 (partially +), CK5/6 (partially +), Ki-67 (80% +), P40 (+), INSM1 (−), POU2F3 (−), CgA (−), Syn (−), CD117 (−) and Vim (+). The cut surface of the left atrial tumor was grayish-yellow, consistent with atrial myxoma.

The postoperative course was satisfactory. The patient recovered well, showing stable improvement without any specific discomfort. Contrast-enhanced CT angiography and 3D reconstruction conducted 4 weeks post-surgery confirmed the complete resection of the masses and without recurrence (Fig. 4C and D). Postoperative echocardiography showed the total removal of the left atrial tumor, with no hemodynamic abnormalities (Fig. 4E). The patient was then referred to the oncology department for further conventional chemoradiotherapy treatment. During the 1- and 3-month follow-up period, no adverse events or unforeseen complications emerged, and no recurrence were found.

## Discussion

Cardiac tumors are infrequently encountered clinical issues characterized by substantial heterogeneity in pathology and clinical presentation. The diagnosis and management of cardiac tumors have been challenging since their discovery in the Middle Ages [1]. Cardiac tumors are classified into primary cardiac tumors, which originate in the heart, and secondary cardiac tumors, which are metastatic tumors that invade the heart. Primary cardiac tumors have a low incidence rate, whereas secondary cardiac tumors are estimated to be a hundred times more common than primary cardiac tumors [2, 3].

90% of all primary cardiac tumors are benign. Myxoma is the most common benign tumor (75%), typically found in the left atrium, followed by the right atrium, and occasionally in the ventricles. Myxoma can present with intra-cavitary obstruction, embolism, and constitutional symptoms, but it may also be asymptomatic and discovered incidentally via echocardiography [4]. Although myxomas have been reported in both genders and all age groups, they are more common in women and individuals in their 60 s [5]. More than 90% of myxomas are sporadic and rarely recur after complete resection [6]. However, the recurrence risk for myxomas is between 1–3%, often associated with multicentric and familial myxomas [7]. Familial incidence accounts for up to 7% of all cardiac myxomas and is linked to Carney complex, an autosomal dominant hereditary condition due to a mutation in the PRKAR1A gene located at 17q24 [11, 12]. In this case, the myxoma originated in the left atrium and was completely removed through surgery.

Secondary cardiac tumors stem from metastases originating outside the heart. According to two reviews on cardiac metastases, the incidence of cardiac metastasis in patients with lung cancer varies from 15 to 35% [6]. The pathways of metastasis can include direct invasion of the pericardium or myocardium, lymphatic spread to the heart, or hematogenous spread entering the pulmonary veins, reaching the left atrium and left ventricle, and subsequently invading the myocardium via the coronary arteries [8]. When cardiac metastasis from lung cancer occurs, it may ultimately cause the patient's death due to its potential to induce atrial fibrillation [9] and sustained ventricular tachycardia [10]. In the biopsy histopathological report of this case, the instance discussed in this article does not involve cardiac metastatic tumors, but rather an unusual coexistence of three non-homologous tumors: a primary cardiac myxoma, lung squamous cell carcinoma, and a thymic cyst.

Lung carcinoma is one of the leading causes of cancer-related deaths, resulting in an estimated 1.4 million fatalities annually worldwide [13]. Non-small-cell lung carcinoma and small-cell lung carcinoma are the two most prevalent types of lung cancer. Non-small-cell lung carcinomas account for over 85% of lung cancer cases and are subdivided into lung adenocarcinomas (50%), lung squamous cell carcinomas (30–40%), and large cell carcinomas [14]. Adenocarcinomas and squamous cell carcinomas present distinct molecular anomalies [15] and are thought to arise from different progenitor cells [16]. Lung squamous cell carcinomas typically arise proximally (from the primary bronchi) [17], with smoking and chronic inflammation being major risk factors [16]. In reported clinical cases, lung squamous cell carcinomas exhibit a significant tendency to metastasize, such as spreading to kidneys [18] and spleen [19]. There have been instances of lung squamous cell carcinoma combined with small cell lung cancer [20], as well as the previously mentioned cardiac metastasis. However, in this case, based on the examination of pathological sections, we found that the patient's lung squamous cell carcinoma and primary cardiac myxoma were two distinct tumors originating from different sources.

Benign thymic cysts are rare conditions, comprising approximately 1% to 3% of anterior mediastinal masses [21]. These cysts are categorized into congenital and acquired types. Congenital cysts are generally simple, unilocular, and possess thin, translucent walls. The thymic tissue attached to these cyst walls is often atrophied and lacks inflammation [22]. Acquired cysts, also termed multilocular thymic cysts, usually have multiple cystic cavities lined by squamous, columnar, or cuboidal epithelium, and are accompanied by acute and chronic inflammation, fibrovascular proliferation, necrosis, hemorrhage, and cholesterol granuloma formation [23]. Some thymic cysts are associated with thymomas, thymic carcinomas, or other malignant tumors, potentially resulting from cystic transformation due to inflammatory processes [24]. Thymic cysts mainly appear in the first decade of life [25], making them very rare in adults. In reported cases, thymic cysts often present with cervical masses, which can cause diagnostic confusion [26]. They may also be associated with systemic conditions like lupus erythematosus and rheumatoid arthritis, and in rare cases, they are linked to myasthenia gravis [27]. In this specific case, the thymic mass was situated in the anterior mediastinum. Microscopic examination of the resected tissue revealed fibrous cyst wall-like structures lined with ciliated columnar epithelium. Thymic tissue was observed around the periphery of the cyst wall.

Cases of pulmonary squamous cell carcinoma, primary cardiac myxoma, and thymic cysts are often reported separately. Additionally, our search indicates that instances of two tumors occurring simultaneously are not unusual. For instance, Wang reported a case of a 73-year-old male diagnosed with papillary fibroelastomas of the tricuspid valve in combination with lung cancer [28]. Thymic cysts have also been documented to evolve into thymic carcinoma, which subsequently metastasizes to the lungs [29].

Following an exhaustive search across major databases such as PubMed, Web of Science, Embase, and Cochrane Library, we found no existing literature that simultaneously reports primary cardiac myxoma, pulmonary squamous cell carcinoma, and thymic cyst in known case reports. Consequently, this case report is the first to document the coexistence of these distinct entities: pulmonary squamous cell carcinoma, primary cardiac myxoma, and thymic cyst. This report may provide valuable insights for future clinical practice.

## Data Availability

Data are available from the authors upon reasonable request.
